# Prion Protein-Specific Antibodies-Development, Modes of Action and Therapeutics Application

**DOI:** 10.3390/v6103719

**Published:** 2014-10-01

**Authors:** Tihana Lenac Rovis, Giuseppe Legname

**Affiliations:** Prion Biology Laboratory, Department of Neuroscience, Scuola Internazionale Superiore di Studi Avanzati (SISSA), Trieste 34100, Italy; E-Mail: tlenac@sissa.it

**Keywords:** prion, PrP, antibody, recombinant antibody, immunotherapy, molecular mechanism

## Abstract

Prion diseases or Transmissible Spongiform Encephalopathies (TSEs) are lethal neurodegenerative disorders involving the misfolding of the host encoded cellular prion protein, PrP^C^. This physiological form of the protein is expressed throughout the body, and it reaches the highest levels in the central nervous system where the pathology occurs. The conversion into the pathogenic isoform denoted as prion or PrP^Sc^ is the key event in prion disorders. Prominent candidates for the treatment of prion diseases are antibodies and their derivatives. Anti-PrP^C^ antibodies are able to clear PrP^Sc^ from cell culture of infected cells. Furthermore, application of anti-PrP^C^ antibodies suppresses prion replication in experimental animal models. Major drawbacks of immunotherapy are immune tolerance, the risks of neurotoxic side effects, limited ability of compounds to cross the blood-brain barrier and their unfavorable pharmacokinetic. The focus of this review is to recapitulate the current understanding of the molecular mechanisms for antibody mediated anti-prion activity. Although relevant for designing immunotherapeutic tools, the characterization of key antibody parameters shaping the molecular mechanism of the PrP^C^ to PrP^Sc^ conversion remains elusive. Moreover, this review illustrates the various attempts towards the development of anti-PrP antibody compounds and discusses therapeutic candidates that modulate PrP expression.

## 1. Introduction

Prion diseases, or Transmissible Spongiform Encephalopathies (TSEs), represent a group of lethal neurodegenerative diseases. In addition to humans, several mammalian species may develop TSE, including Bovine Spongioform Encelopathy (BSE) in cattle, scrapie in sheep and goat or Chronic Wasting Diseases (CWD) in deer, moose and elk [1]. Certain types of the disease can be transmitted from human to human, such as Kuru or iatrogenic CJD (iCJD); but also from animals to humans, where the most prominent example is BSE in variant Cruetzfeldt-Jacob Disease (vCJD), mostly acquired through the consumption of BSE-infected food. However, less than 5% of prion-caused diseases are acquired, 10% to 15% are defined as genetic, while the remaining majority are considered sporadic [2]. Genetic types of the disease in humans are familial CJD (fCJD), fatal familial insomnia (FFI), prion protein cerebral angiopathy (PrP CAA) and Gerstmann–Sträussler–Scheinker syndrome (GSS), while sporadic types include sporadic CJD (sCJD), sporadic fatal insomnia and variably protease-sensitive prionopathy (VPSPr) [3], the most recently identified prionopathy [4].

These diseases have a long asymptomatic incubation period and largely differ in their clinical course, which typically ranges from a few months to several years. What is common is that all are triggered by misfolding of a host encoded cellular prion protein (PrP^C^) [5]. All TSEs share common neurodegenerative features: aggregation of the misfolded PrP^C^, early synaptic dysfunction and irreversible death of neurons [6]. PrP^C^ is physiologically expressed throughout the body and is highly expressed in the central (CNS) and peripheral (PNS) nervous system, as a normal part of the neuronal membrane. It has a complex intracellular trafficking that seems to depend on the cell type [7]. The development of TSE includes the pathological conversion of the PrP^C^ into the toxic and infectious isoform denoted as prion or PrP^Sc^. PrP^Sc^ faithfully replicates, aggregates and deposits in brain parenchyma and is not prone to degradation via cellular proteases [1]. From the infected cell, horizontal and vertical transmission can occur, since misfolded proteins are efficiently transmitted to the daughter cells and by the intercellular spread [8]. 

The transgenic mice lacking *Prnp* gene are resistant to prion diseases [9] suggesting that the disease progression is dependent on a pool of PrP^C^ within the cell that can be replicated. The PrP knockout mice show no significant phenotype. Likewise, the conditional *Prnp* knockout showed no signs of neurodegeneration [10]. This focused the design of therapeutic approaches towards the attenuation of PrP^C^ [11]. However, a growing body of data reveals potential physiological PrP^C^ functions, including its neuroprotective role in the CNS, while the loss of PrP^C^ function renders the cells more susceptible to different types of stress [12]. In spite of this, the lack of deleterious effects upon the absence or silencing of PrP, observed in relevant animal models, infers a window of opportunity that can be used for the treatment aimed at the neutralization or depletion of the PrP^C^. This review will focus on the role of prion-specific antibodies in the modulation of PrP biology and the development of related therapeutic applications. 

## 2. Therapeutic Candidates that Modulate PrP^C^ Expression or Accessibility to Conversion

A number of drugs have been tested for therapeutic intervention in patients affected by TSEs, but none significantly increase the survival of patients [13]. The hypothesis that PrP^C^ is essential for prion replication, but dispensable for the host, resulted in two types of anti-prion compounds that target PrP^C^ expression.

First, some drugs have been tested that are considered safe for human health, and possess the desired ability to modulate PrP^C^ expression, either by reducing or rearranging its cellular pool. A prominent example is suramin [14] and its derivatives which modulate biochemical properties of PrP^C^ including solubility, its half-life [15] and, according to other studies, internalization rate [16]. Another example of a PrP^C^ modulator that inhibits formation of the scrapie isoform is the drug mevinolin [17], which has multiple generic names and is used to lower cholesterol [18]. Mevinolin reduces the surface expression of PrP^C^ leading to its intracellular accumulation [19]. Tamoxifen, another pharmaceutical [20], and its derivative 4-hydroxytamoxifen were recently shown to redirect cholesterol to lysosomes and consequently induce PrP^C^ as well as PrP^Sc^ degradation through enhanced lysosomal trafficking and degradation [21]. However, a list of chemotherapeutics targeting PrP^C^ expression, PrP^Sc^ expression or the conversion, including pentosan polysulfate, quinacrine, amphotericine B and flupirtine, have already been tried in clinical trials showing no or modest treatment efficacies [22]. Recently, a comprehensive drug screening was undertaken to identify new anti-PrP agents among drugs already approved for human use [23]. Screening targeted compounds that decrease PrP^C^ expression. The most promising candidate, astemizole, prolonged the survival of prion-infected mice via stimulated autophagy [23].

**Figure 1:**
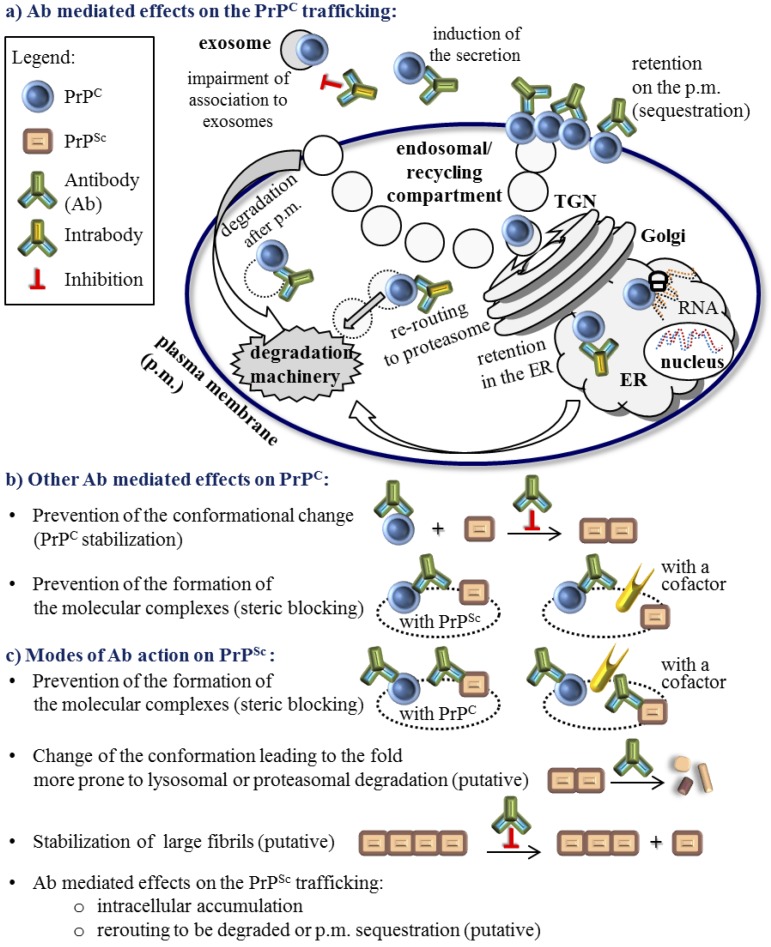
Anti-prion protein antibody (Ab) modulation of the PrP^C^ and PrP^Sc^ biology. There are no documented Ab effects on the PrP^C^ transcription or translation. Ab effect on the immature PrP^C^ is restricted to some intrabodies. (**a**) Published Ab mediated effects on the PrP^C^ trafficking; (**b**) Other modes of Ab impact on mature PrP^C^ that do not include the PrP^C^ trafficking modulation and are not restricted to the specific compartment; (**c**) Modes of Ab action on the PrP^Sc^ cannot be easily separated from the modulation of PrP^C^ because the percentage of the PrP^Sc^ in the cell is much lower and most of the Abs that recognize PrP^Sc^ do not discriminate between forms. Not fully confirmed modes of action are noted as ‘putative’. Undefined cellular compartments are depicted with a dotted line. Full IgGs and other recombinant Abs (Figure 2), except for intrabodies, are schematically represented by the same Ab symbol.

The second line of compounds specifically target PrP^C^ and as such their mode of action in principle should not affect other aspects of cellular biology, including the cell viability. One straightforward approach to specifically decrease PrP^C^ levels is to target the expression of the gene responsible, in humans *PRNP*, either with interfering RNA molecules or by introducing a dominant negative mutant [24]. Molecules that bind specifically to PrP^C^ include nucleic acid aptamers and peptide aptamers [25], which show inhibitory effect on prion conversion. In addition, a broad range of evidence shows that antibodies targeting PrP^C^, as a template for the scrapie prion propagation, are effective in curing infected cells [26,27,28,29]. Anti-PrP^C^ antibodies and their derivatives represent a range of compounds able to reduce availability of the PrP^C^ substrate for conversion; either by minimizing PrP^C^ expression and inducing its redistribution from the sites critical for prion conversion or by preventing the formation of the molecular complexes between PrP^C^ and PrP^Sc^ and other potential cofactors (Figure 1 and discussed below). In addition, antibodies may act as other potential drugs that bind PrP^C^ and tend to stabilize the PrP^C^ molecule in order to prevent conversion [11]. 

Beyond PrP^C^ many drugs target PrP^Sc^ template. The awareness of the need for combination therapy is evolving after the anti-prion drug resistance was confirmed [30] for most promising candidates obtained within a comprehensive study of more than 10,000 small molecules able to reduce PrP^Sc^ content [31]. Recently, a new battery of promising small molecules with the ability to decrease the accumulation of PrP^Sc^ was obtained in another comprehensive screening that evaluated their drug ability and pharmacokinetic parameters [32]. When targeting PrP^Sc^ template, the aim may be to promote degradation, as observed for some branched polyamines [33] or for tyrosine kinase inhibitor STI571, which promotes lysosomal degradation [34]. Alternatively, the aim may be to stabilize fibrils as was proposed for congo red [35] or luminescent conjugated polymers [36] in order to reduce the spread of low molecular weight oligomers that seem to be particularly infectious and toxic [37,38].

Antibodies so far have not been implied in the stabilization of PrP^Sc^, but in principle they could be able to do so if they recognized epitopes of the PrP^Sc^ amyloid fibrils (Figure 1). Alternatively, antibodies can selectively target misfolded proteins while sparing native, properly folded protein [39,40,41,42,43,44,45]. An antibody that would bind specifically to PrP^Sc^ could modulate PrP^Sc^ trafficking or inhibit PrP^Sc^ interaction with other molecules analogously as described for PrP^c^ (Figure 1). Some studies found that PrP^Sc^ recognition is a beneficial feature of a curing antibody [46]. However, a significant number of curing antibodies do not recognize PrP^Sc^ [28]. In addition, some antibodies that recognize PrP^Sc^ have weak curing properties when administrated into *in vivo* or *in vitro* settings [27,45,47,48]. 

In conclusion, antibodies and their derivatives are on the list of most prominent candidates for the treatment of prion diseases [49,50] due to their effectiveness at targeting the PrP^C^ as a reservoir for the prion conversion but also because of their potential to act on multiple and diverse levels in the prion pathogenesis.

## 3. The Role of Antibodies in the Molecular Mechanism of the PrP^C^ to PrP^Sc^ Conversion

The key process behind prion diseases is the conversion of PrP^C^ into the PrP^Sc^ isoform. In this process anti-PrP antibodies represent one of the most promising strategies for the treatment of prion diseases ever since they not only reduced, but completely cleared the pre-existing PrP^Sc^ from a culture of infected neuroblastoma cells [26,29,51]. However, the molecular mechanisms behind the conversion of PrP^C^ into PrP^Sc^ and the role anti-PrP antibodies play remains elusive. 

Regarding some aspects of the antibody-mediated process of clearing PrP^Sc^ a general consensus has been reached. There is a direct correlation between the affinity of anti-PrP antibody for the PrP^C^ isoform and its potency to cure prion infected cells [27,28,52]. Furthermore, there is no unique epitope in the PrP^C^ molecule that clears the disease, although not all epitopes are suitable or equally effective [28,47,52,53,54,55]. Finally, the compartment(s) of prion conversion are still the matter of debate, but the lines of evidence [56,57,58,59,60,61] including the most recent studies [62,63,64,65] largely agree that the plasma membrane and the membrane trafficking along the endocytic-recycling pathway are prominent sites where PrP^C^ and PrP^Sc^ reside. Such localization favors the accessibility of both targets to the antibodies and warrants the maintenance of the stable antibody-target complexes. The ability of an antibody to recognize native PrP^C^ molecules expressed on the plasma membrane may discriminate protective *vs.* non-protective immune responses [66,67,68]. In many cases, antibodies that have shown significant clearing capacities were internalized into the cells [28,52,69,70] suggesting their potential to exhibit additional positive effects also along the endocytic pathway.

Despite the convergence of several important issues of the antibody clearing capacity, there are a high number of mechanisms proposed for the function of anti-PrP antibodies. It is evident that these molecular mechanisms depend on the antibody epitope and in addition, different mechanisms do not necessarily exclude one another. Among them are: steric blocking or modifying the interaction of PrP^C^ with PrP^Sc^ [26,52,71]; perturbation of PrP^C^ trafficking, including internalization and degradation [28,52,70,72,73]; PrP sequestration on the cell surface [28,29,52,54]; increase of PrP^C^ levels in the medium [28,52]; and neutralization of the infectious PrP^Sc^ template [52,72]. 

Although relevant for designing immunotherapeutic tools, the characterization of the antibody role in PrP^C^ conversion to PrP^Sc^ is still not fully clarified. Thorough understanding of this molecular mechanism will contribute to the design of anti-prion therapeutics and to general principles of immunotherapy. 

**Figure 2:**
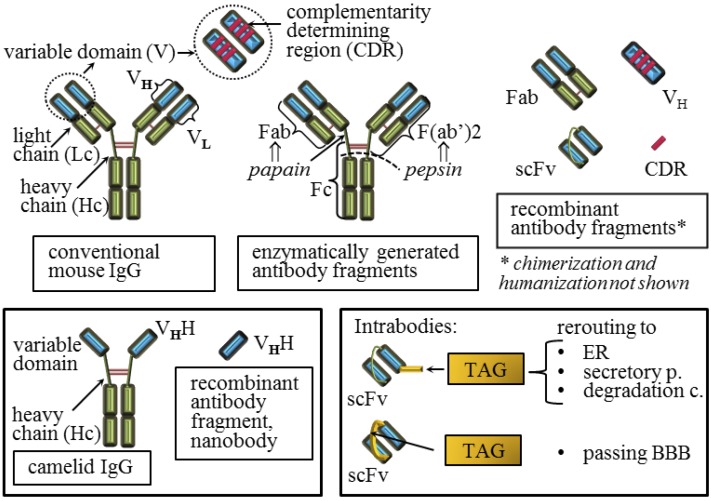
Conventional and recombinant antibody compounds that have been developed against the prion protein. Schematic representation of mouse natural IgG (conventional IgG) that is composed of two light chains and two heavy chains linked together with disulfide bridges is shown. A variable domain of the conventional antibody that binds to the specific antigen is composed of the sequence on the heavy and on the light chain while each of these sequences is composed of three complementarity determining regions (CDRs). Common IgG fragments generated by enzymatic digestions or by the recombinant DNA technique are shown. The natural camelid IgG possess only two chains linked by disulfide bridges and each variable domain is composed of a single chain. Intrabodies are recombinant intracellular antibodies that are usually engineered to localize to a specific cellular compartment.

## 4. Active and Passive Immunotherapy Approaches

Active immunotherapy implies the production of anti-PrP antibodies by the host, mostly following vaccination, while in the passive immunotherapy a pre-made therapeutic, an antibody-based compound, is delivered directly or by the gene therapy.

Active immunotherapy suffers from the tolerance of the immune system to develop antibodies to the host protein. The prion protein is a native cellular protein and an organism is unlikely to recognize the subtle changes attributed to PrP^Sc^ as a threat especially as it is imprinted in the immune system to avoid the self-recognition. A number of approaches were undertaken in active immunization studies, including various antigens and adjuvants in order to break the tolerance against PrP [49,50]. The evaluation of essential protective immune response properties in different mouse models revealed the importance of an antigen to provoke antibodies recognizing cell-surface PrP^C^ [66]. This finding was confirmed in the recent study identifying compounds with the best immunogenic potential, in which the protection model was further associated with depletion of mature follicular dendritic cells, which spread peripheral prion infection [67]. Encouraging results in the generation of host antibodies towards PrP^C^ were obtained using PrP monomer peptides, multiple antigenic peptides, full PrPs, proteins resembling PrP epitopes or PrP dimers as the antigen of choice, administrated through various vector-, protein-, virus- or cell-carriers [49,50,74]. In addition, naturally occurring PrP autoantibodies were recently confirmed in cerebrospinal fluid and serum samples of healthy individuals [75]. The approach to target the PrP^Sc^ template and at the same time avoid the recognition of abundantly expressed PrP^c^, guided the development of the vaccine based on the PrP^Sc^ dominant epitope YYR [40]. The follow up studies were focused to (i) exclude the concern that such anti-PrP^Sc^ antibodies might by themselves induce the formation of the PrP^Sc^ template and to (ii) drive the anti-PrP antibody response towards conformations of PrP mutants associated with genetic types of the disease [76]. The animal studies show that still mucosal vaccination seems to be the most effective, although this approach would be restricted to preventing oral transmission among animals and human populations at risk [77,78]. 

Ubiquitous PrP^C^ expression not only aggravates the natural expansion of anti-PrP antibodies, but also the introduction of premade antibodies recognizing PrP^C^ via passive immunotherapeutic approaches might lead to severe immune reactions. This concern was alleviated after the groundbreaking study showing that co-expression of PrP-specific antibodies with PrP^C^ expressed at physiological levels does not induce autoimmune responses or significant changes in various immune cell populations, which were still able to respond to other stimuli [71]. At the same time, the substantial anti-PrP antibody levels in the serum prevented scrapie pathogenesis after prion inoculation. Another study, based on subsequent and continuous passive immunizations initiated immediately following the scrapie inoculation, resulted in the prolongation of the incubation period or even prevention of disease, depending on the antibody used and the inoculum quantity [79]. The passive immunization attracted further interest after it was shown that the application of anti-PrP antibodies suppressed, in some cases even permanently, a peripheral prion replication *in vivo* and that such treatment was successful even after the onset of peripheral prion replication, although not after the clinical signs of illness were present [47]. In addition, administrated anti-PrP antibodies did not deplete PrP expressing immune cell populations nor was evidence for autoimmune reactions found. Recent data showed that in addition to intraventricular administration [80], a peripheral administration of antibodies could alleviate the disease progression also at the time of clinical onset [81]. The efficacy of the antibody distribution in the cerebella and thalami was in the correlation with the prolongation of survival times. Indeed, the delivery of the therapeutic to the infected brain is essential and as such new vectors that allow for the better delivery of anti-prion protein antibodies into the brain are being envisaged (discussed below, [82,83,84]). Likewise, recombinant antibody-derivatives with improved drug characteristics are being designed (discussed below). However, advancements of the numerous passive immunizations in the last decade [49] that resulted in more or less significant increase in the resistance to PrP^Sc^, including the prolongation of the incubation period and the lifespan of treated animals, have been compromised with their possible neurotoxic effects [55,85]. These effects are still a matter of debate [86], but certainly pose additional requests to the design of the antibody-based therapeutics. 

A line of evidence shows how PrP^C^ plays an important role in the pathogenesis of other neurodegenerative diseases, such as Alzheimer’s disease (AD) [87]. Among neurodegenerative disorders, clinical development of immunotherapeutic strategies to cure AD patients is by far most advanced [88]. Unfortunately, active anti-AD immunization trials were stopped due to the severe side effects [88] while two phase III trials of anti-β-amyloid monoclonal antibody recently showed that passive immunotherapy provokes less alarming side effects, but does not improve clinical outcomes in patients with AD [89]. Regardless, new links between distinct neurodegenerative diseases will undoubtedly boost the anti-prion immunotherapeutic approaches including the especially relevant topic of better understanding of toxic side effects. A prominent example is the recent study on fully humanized anti-PrP antibody that was shown to prevent Aβ synaptotoxicity in rats without inducing obvious neurotoxicity [90]. As already mentioned, our understanding of the molecular mechanisms behind the mode of action of anti-PrP antibodies is insufficient and, correspondingly, multiple mechanisms are still proposed for the antibody-mediated plaque removal in AD [88]. The findings from the research on fundamental principles driving the immunotherapy of prion illnesses may thus provide a breakthrough in our knowledge of the more common neurodegenerative diseases.

## 5. Molecular Parameters that Influence the Quality of the Anti-Prion Protein Antibody Effect

In spite of the continuous progress, the major drawbacks of the passive immunization approach are still the (i) unfavorable pharmacokinetic of drugs; (ii) high amount of the drugs needed; (iii) and the inability of drugs to cross the blood-brain barrier (BBB) if they are not focused on the inhibition of peripheral prion replication and must access the central nervous system (CNS); all the above probably lead to limited success as therapeutics *in vivo*. Last but not the least; (iv) there is a concern that anti-PrP^C^ antibodies might be neurotoxic. To that aim different recombinant antibody derivatives with different properties have been designed. Still, clinical immunotherapy trials in the neurodegenerative diseases used conventional full length IgG, although in the recombinant, humanized form [89]. In spite of the general complaint of IgG low potential to reach the CNS, *in vivo* studies showed that autoantibodies against Aβ can in fact cross the BBB [49] and peripheral administration of humanized form of IgG reached therapeutically active concentrations to prevent Aβ synaptotoxicity [90].

Since the generation of the first immunogens, numerous anti-PrP antibodies and antibody compounds have been developed, at first polyclonal [91] and later mostly by the immunization of *Prnp*°^/^° mice [9]. Interestingly, the first antibodies obtained upon the immunization of *Prnp*°^/^° mice were generated in the active form of the recombinant antigen-binding fragment, Fab (fragment antigen-binding), by the phage display technology due to the instability of initially acquired hybridoma cell lines secreting conventional monoclonal antibodies (Figure 2) [92]. Series of conventional antibodies have been raised since then, with promising candidates able to cure PrP^Sc^ *in vitro* with half maximal inhibitory concentration of PrP^Sc^ (IC50) far below 1 μM [50]. The process of developing new panels of anti-PrP antibodies is still in progress [45,93,94,95,96]. A study aimed at characterizing the pharmacokinetic properties of anti-PrP antibodies with curing properties *in vitro* showed that their curing capacity *in vivo* is associated with intrinsic pharmacokinetic properties rather than their isotype, epitope or affinity [97].

Recombinant Fab fragments (Figure 2) successfully cleared prion infectivity from cell cultures of infected cells [26]. Although a difference between polyclonal IgG molecules and corresponding Fab fragments in their capacity to inhibit prion replication in infected cells was observed [51], a comparison of several full IgGs and their Fab fragments showed that they retain similar binding properties and similar curing capacity [98]. The Fab fragments should be less prone to induce neurotoxicity, but this is still disputed. Namely, two studies showing neurotoxic side effects upon the antibody injection into the brain agreed that antibody mediated neurotoxicity was not mediated by its Fc (fragment crystallizable) fragment, but by triggering PrP^C^ and that toxicity was dependent on the dosage of the antibody treatment [55,85]. Unfortunately, these studies did not reach agreement about other antibody parameters that should be taken into consideration during drug development nor on the molecular mechanism triggering downstream neurotoxic effects. The first study proposed that the divalent antibody form is responsible for crosslinking and triggering PrP^C^ molecules leading to cell apoptosis [85], while the other proposed that the antibody epitope within a particular PrP^C^ domain is detrimental for calpain activation [55]. The latter study showed no significant differences between the monovalent and divalent forms of the antibodies tested. Fortunately, in both studies some antibodies escaped the neurotoxic phenotype. Taken together with studies showing no deleterious effects upon the comparable antibody administration [54,80,86], emphasizes that generalizations about the toxicity of antibodies should be avoided and strongly suggests that a therapeutic window must exist. Certainly, Fab fragments have shorter circulating half-lives, but improved production opportunities via recombinant prokaryotic expression and enhanced capacity of penetrating into the brain [98,99] when compared to the full IgGs. However, in respect to these later advantages, smaller recombinant compounds are even more promising (Figure 2, [100]) and in addition to the Fabs and IgGs, many smaller monovalent compounds have been designed.

The limitations of full IgGs: the poor influx into CNS, the putative toxicity and the complex assembly; prompted studies aimed at generation of more potent recombinant proteins on the backbone of antibodies with desirable affinity characteristics. In line with that, recombinant anti-PrP scFvs (single-chain variable fragments, Figure 2) were designed and verified in neurodegenerative disease models [101]. Here, the scFvs retained the ability to clear the PrP^Sc^ infected cell cultures [68,84,102,103,104]. Recently, a humanized anti-PrP^Sc^ scFv has been produced [105]. The acknowledged advantage of the scFvs is their expression, suitable for large scale production in the periplasmic space of *E. coli.* In parallel, several eukaryotic cell lines secreting scFvs have been established [68,102,103]. One of the trasnsfectants was made on the background of immortalized microglia, acknowledged brain-engraftable cells that resulted in a short prolongation of the survival times in mice [103]. Indeed, the main advantage of the scFv is its single polypeptide sequence suitable for the gene transfer-based passive immunization, the approach in which the antibody is not delivered by the direct application, but by a corresponding gene encoding the antibody later synthesized by the host. The most recognized vectors for the delivery of these antibody genes, possessing high transduction efficiency and allowing intracerebral spread, are adeno-associated virus (AAV) based vectors. Two studies on vector types AAV2 and AAV9, both carrying genes for anti-PrP scFv, have resulted in a delay in the onset of clinical signs of disease, prolonged survival time, milder neuropathological changes, reduced PrP^Sc^ burden in the brain and, importantly, no inflammatory or neurotoxic effects together with prominent neuronal transduction efficiency and spread [82,83]. However, these beneficial outcomes were not all significant and both groups in their experimental model used intracerebral injection of the vectors followed by intraperitoneal challenge at expected peak of the scFv gene expression. A study aimed to design molecules and delivery mode that might function both peripherally and within the brain, thus affecting both sites of prion replication, explored the potential of lentiviral and AAV vectors encoding anti-PrP scFv [84]. In cell culture models of PrP^Sc^ clearance the lentiviral construct represented a more efficient delivery system compared to AAV. The scFv antibody format is also prevalent for the intracellular antibody (intrabody) expression [73]. These recombinant antibodies are engineered to localize to a specific cellular compartment. The Anti-PrP scFv with ER retention signal successfully retained PrP^C^ in the ER and prevented PrP^Sc^ formation in the corresponding transfected cell lines, while the secretory version of the same intrabody mediated re-routing of PrP^C^ to proteasome as well as impairment of PrP^C^ association to exosomes [73]. An interesting scFv was recently obtained by fusing anti-PrP^Sc^ antibody variable domains with an advanced linker, cell-penetrating peptide (CPP), penetratin [106]. Upon administration in the mouse tail vein, the scFv without penetratin mainly stained the endothelial cells of brain veins, while the penetratin-scFv was transported through the BBB into the brain cells. However, an unexpected localization into the nuclei was observed that might necessitate additional modifications of this promising recombinant antibody.

In addition to the scFvs, other, smaller antibody forms have been envisaged. To that aim, camelid antibodies are of great interest, since they lack light chains and consequently possess a genuine single chain variable domain. Thus, corresponding recombinant antibody fragments, called nanobodies, are significantly smaller than scFvs obtained from the conventional antibodies (Figure 2). The ability of recombinant camelid antibody fragments to abolish prion replication in infected cell lines [72,96] and to diffuse into the brain parenchyma upon peripheral administration was confirmed [69]. A variable domain of the conventional antibody is composed of the sequence on the heavy and on the light chain and each of these sequences is composed of three complementarity determining regions (CDRs, Figure 2). The heavy chain of the anti-PrP antibody, combined with unrelated light chains, retained the capacity to prevent prion pathogenesis upon peripheral scrapie challenge [71]. Furthermore, a peptide mimicking only the third CDR of the anti-PrP heavy chain domain (CDR3H) showed anti-prion capacity *in vitro* [104]. 

Many promising anti-PrP antibody compounds have been produced so far. The main concern remains that, unless an artificial amount of an antibody is supplied to the site of infection, the reduction of the PrP^c^ content in patients might only postpone and not prevent the illness [107]. The fact that a small amount of PrP^C^ is enough for the productive replication underscores the hypothesis [108]. Alike, the need for the improvement of diagnostic tools that could pinpoint the illness at the earliest stage goes hand in hand with the need to optimize the infection::antibody ratio. Most of the administrated antibody compounds, including the full IgGs, do not have suitable characteristics to cure the infection in brain, but at the same time the gene-based delivery routes are still providing only a short term supply [82,83,103]. Although our future might decide on the gene therapy with the smallest possible antibody based drugs, currently in the treatment of neurodegenerative diseases conventional humanized IgG approach is still mainstream.

## 6. Authors’ Perspective

For more than a decade, the scientific community has been trying to envisage innovative therapeutics based on the anti-prion protein antibodies and new vaccines able to break immune tolerance against the prion protein. A remarkable pool of structural data and a considerable list of antibodies and recombinant antibody-forms generated to a single protein, the prion protein, offer a unique possibility to explore the fundamental premises of the immunotherapy. Unfortunately, few studies compared the original antibodies and their recombinant derivatives or a palette of recombinant antibodies recognizing the same epitope in thorough *in vitro* or *in vivo* studies. The influence of the size/form/valency/posttranslational modification of the antibody derivatives on the fundamental molecular mechanism triggered by their binding to the PrP molecules is still elusive. Among others, this includes the factor of antibody size on steric hindrance and blocking of PrP^C^ conversion, the capacity of different derivatives to be internalized into the cells, the importance of their ability to crosslink the PrP molecules and induce or block endocytosis, the antibody-PrP complexes’ stability and dissociation of PrP molecules within various organelles and the molecular determinants triggering neurotoxic effects. In addition, the antibody glycosylation is very complex and its influence on the subtle differences in the antibody mode of action will be an interesting target for examination. Once rules that are more general with respect to the molecular mechanisms and the drug characteristics influencing PrP^Sc^ clearance are established, it will be easier to manipulate the functional and curative anti-PrP antibody properties. Such scientific outcomes will contribute to the understanding of general principles of recombinant antibody design and immunotherapy.
